# Maternal and Cord Blood Hemoglobin as Determinants of Placental Weight: A Cross-Sectional Study

**DOI:** 10.3390/jcm10050997

**Published:** 2021-03-02

**Authors:** Ferrante S. Gragasin, Maria B. Ospina, Jesus Serrano-Lomelin, Su Hwan Kim, Matthew Kokotilo, Andrew G. Woodman, Stephen J. Renaud, Stephane L. Bourque

**Affiliations:** 1 Department of Anesthesiology & Pain Medicine, University of Alberta, Edmonton, AB T6G 2G3, Canada; gragasin@ualberta.ca (F.S.G.); msk1@ualberta.ca (M.K.); 2 Women and Children’s Health Research Institute, University of Alberta, Edmonton, AB T6G 1C9, Canada; mospina@ualberta.ca (M.B.O.); jaserran@ualberta.ca (J.S.-L.); agwoodma@ualberta.ca (A.G.W.); 3 Department of Obstetrics & Gynecology, University of Alberta, Edmonton, AB T6G 2R7, Canada; 4 Department of Mathematical & Statistical Sciences, University of Alberta, Edmonton, AB T6G 2G1, Canada; suhwan@ualberta.ca; 5 Department of Pharmacology, University of Alberta, Edmonton, AB T6G 2H7, Canada; 6 Department of Anatomy and Cell Biology, University of Western Ontario, London, ON N6A 5C1, Canada; srenaud4@uwo.ca

**Keywords:** anemia, fetal development, hemoglobin, placenta, pregnancy

## Abstract

Background: Both high and low placental weights are associated with adverse pregnancy outcomes. Maternal hemoglobin levels can influence placental weight, but the evidence is conflicting. Since maternal hemoglobin does not invariably correlate with fetal/neonatal blood hemoglobin levels, we sought to determine whether cord blood hemoglobin or maternal hemoglobin status more closely associates with placental weight in women undergoing elective cesarean section at term. Methods: This was a cross-sectional study conducted at the Royal Alexandra Hospital, Edmonton, Canada, involving 202 women with term singleton pregnancies undergoing elective cesarean section. Maternal blood and mixed cord blood hemoglobin levels were analyzed using a HemoCue Hb201+ system. Birth weight, placental weight, one- and five-minute APGAR scores, American Society of Anesthesiologists physical state classification, maternal age, and maternal height were also recorded. Relationships between maternal and cord blood hemoglobin levels with placental weight, birth weight, and birth weight to placental weight ratio were the main outcome measures. Results: A total of 182 subjects were included in the analysis. Regression analysis showed that cord blood hemoglobin, but not maternal hemoglobin, was inversely related with placental weight (*β* = −2.4, *p* = 0.001) and positively related with the birth weight to placental weight ratio (*β* = 0.015, *p* = 0.001 and *p* = 0.63, respectively). Conclusions: Our findings suggest that measuring cord blood hemoglobin levels, rather than maternal hemoglobin levels, may provide important diagnostic information about in utero fetal adaptation to suboptimal placental function and neonatal health.

## 1. Introduction

Optimal fetal growth and development are dependent on an adequate supply of oxygen and nutrients, which is obtained from the mother via the placenta. When a mismatch occurs between placental supply and fetal demand, morphological and functional adaptations by the placenta can reduce the incidence of fetal growth restriction or macrosomia [1]. By extension, pregnancies characterized by inadequate placental adaptation can result in aberrant fetal growth with implications for long-term health. For example, small for gestational age babies have smaller placentas compared to those born appropriate for gestational age [2], suggesting that a failure of the placenta to grow and adapt to meet the needs of the fetus results in growth restriction. Many factors have been reported to influence placental size and weight, including anemia [3,4]. Anemia is characterized by reduced blood hemoglobin (Hb) levels and reduced oxygen-carrying capacity in the blood. It is a common health complication in pregnancy, most often attributed to iron deficiency due to increased iron demands from the growing conceptus and maternal blood volume expansion [5]. In animal models, maternal anemia, in conjunction with fetal anemia, impacts oxygen delivery to fetal tissues, alters offspring growth trajectories, and results in markedly bigger placentas [6,7]. In humans, maternal anemia is associated with enlarged placentas [8,9,10], high placental to birth weight ratios [3,11], and increased placental vascularization [12,13]—morphological changes that may reflect adaptation to improve oxygen delivery in the wake of reduced Hb levels. However, other studies have reported no effect of maternal anemia on placental weight [12,14,15], or even reduced placental weight with maternal anemia [16,17]. Thus, the relationship between maternal Hb status and placental weight may be more complex and depend on additional factors such as the gestational age of anemia onset and whether the placenta and fetus maintained an appropriate level of Hb to sustain adequate oxygen delivery and growth.

Fetal blood Hb is inherently difficult to measure, so it is rarely assessed. Consequently, fetal Hb status is typically estimated based on maternal Hb levels, with the implicit assumption that the two indices are correlated. Our recent study found that indices of maternal iron status (e.g., serum ferritin) correlate poorly with fetal levels [18], and the relationship between maternal and fetal Hb is also variable [18], in some cases showing moderate to strong correlation [19,20,21,22] and others none [23,24]. Since anemia, per se, drives placental compensation rather than iron deficiency [11], the question arises as to whether maternal or fetal Hb status is a more important predictor of placental size. The objective herein was to investigate whether fetal (i.e., cord blood) or maternal Hb levels are more closely associated with placental weight, birth weight, and birth weight to placental weight ratio.

## 2. Materials and Methods

This was a clinical cross-sectional study of singleton pregnancies who underwent elective cesarean sections at the Royal Alexandra Hospital in Edmonton, Canada, between January 2016 and November 2017. Reasons for undergoing elective cesarean sections included patient request, repeat cesarean sections, small pelvic outlet relative to the size of the baby, breech presentation, or request for tubal ligation at delivery. Patients were consented prior to elective cesarean section. Women were eligible for the study if they were >18 years of age and were Class I to III, according to the American Society of Anesthesiologists (ASA) physical status classification (assessed by the attending anesthesiologist). Exclusion criteria were: non-elective cesarean section, presence of vaginal or intrauterine bleeding due to placenta pathology (i.e., placenta abruption, placenta previa, placenta accreta, placenta increta, and placenta percreta), or a cesarean section requiring general anesthesia.

After consent was obtained, spinal anesthesia was instituted, and once confirmed to be adequate, maternal Hb levels were assessed from a toe prick using a HemoCue Hb 201+ system. Upon delivery of the fetus, the umbilical cord was clamped, and a sample of mixed cord blood was collected by the surgical team prior to delivery of the placenta; cord blood was collected in a small cuvette, and Hb level was assessed using the same HemoCue Hb 201+ system. The untrimmed placenta (i.e., including membranes and residual blood) was then placed in a plastic bag and weighed (in grams). Birth weight was measured in grams, and the ratio of birth weight to placenta weight was calculated. Additional information collected from participants’ medical charts included maternal height, maternal weight, gestational age of fetus at time of birth (assessed by last menstrual period, unless uncertain, and then assessed by ultrasound), sex of the neonate, one- and five-minute APGAR scores (based on neonatal nurse’s assessments), and maternal ASA class (as per attending anesthesiologists’ assessment at the time of surgery). Ethics approval was obtained from the University of Alberta Health Research Ethics Board (Pro00059156). No confidential information was recorded; subjects were identified by date of surgery and subject number in ascending order for the day.

Exploratory statistical analysis was conducted to characterize the variables’ distribution and to detect outliers. Categorical variables are reported as frequencies and percentages, and continuous variables are reported as means and standard deviations (SD). Pearson’s coefficients (r) were calculated for bivariate correlation analysis to explore and describe linear relationships among the continuous variables. Multiple linear regression models were used to predict placental weight, birth weight, and birth weight to placental weight ratio (main outcomes), based on maternal and cord blood Hb levels, adjusting for important covariates (e.g., maternal age, maternal height, ASA class, gestational age, infant sex, and APGAR score) as explanatory variables. The Shapiro-Wilk test was used for testing the normality of variables. Due to the exploratory nature of our study, all explanatory variables in the models, except for the ones highly correlated with reducing collinearity, were included. Graphs of the residuals were analyzed to validate model assumptions. All statistical analyses were conducted in Stata-IC (version 15.0, Stata Statistical Software, College Station, TX). The figure was generated using Prism software (version 8.0, GraphPad, San Diego, CA, USA).

## 3. Results

A total of 202 women consented to participate in the study. Of these, a total of 20 women were excluded; 12 were excluded due to incomplete data collection (e.g., neonatal sex was not recorded), and eight were excluded due to recording errors, leaving a total of 182 uncomplicated singleton pregnancies included in the analysis. Characteristics of subjects included in the study are shown in Table 1. The mean age was 32.5 years. Mean maternal Hb level was 111.5 g/L; 47 subjects (26%) met the criterion for mild anemia (Hb: 100–110 g/L), 32 subjects (18%) for moderate anemia (Hb: 70–99 g/L), and none (0%) for severe anemia (Hb: < 70 g/L). [25] Mean cord blood Hb was 142.3 g/L. Two neonates met the criterion for low birth weight (at 2400 g and 2430 g) but had normal Hb levels (146 and 176 g/L, respectively).

**Figure 1:**
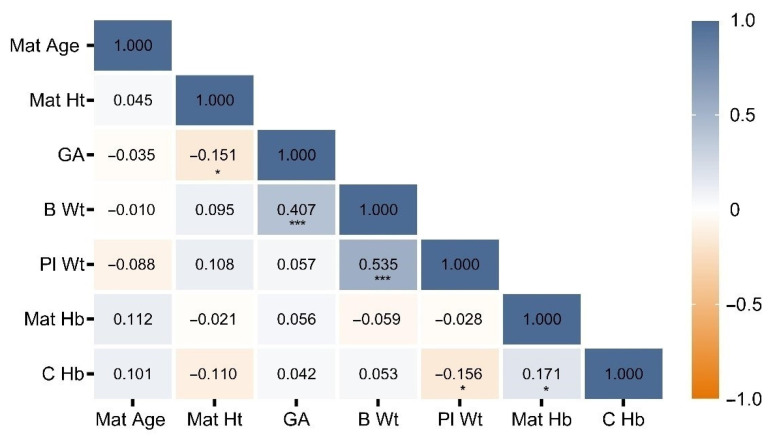
Correlation matrix showing Pearson’s coefficients of the bivariate (unadjusted) analysis. * *p* < 0.05, *** *p* < 0.01. B, birth; C, cord; GA, gestational age; Hb, hemoglobin; Ht, height; Mat, maternal; Pl, placental; Wt, weight.

Results of the bivariate correlation analysis are shown in Figure 1. Strong positive correlations were found between birth weight and placental weight (*p* < 0.001) and gestational age and birth weight (*p* < 0.001). A negative correlation between cord blood Hb and placental weight was observed (*p* = 0.036), as was a positive correlation between cord blood and maternal Hb levels (*p* = 0.021).

Multivariate linear regression analysis was conducted with placental weight as the primary outcome (Table 2). Birth weight (*p* < 0.001), cord blood Hb levels (*p* = 0.001), and gestational age (*p* = 0.005) were strong predictors of placental weight, whereas maternal Hb was not (*p* = 0.428). For every unit (g/L) decrease in cord blood Hb, meant placental weight increased by 2.4g while all other variables in the model were kept constant. The model, using these variables, explains 38% of the variance in this dataset.

Multiple linear regression analysis with birth weight as the primary outcome is shown in Table 3. Only gestational age (*p* < 0.001) and placental weight (*p* < 0.001) were predictors of birth weight; neither cord blood Hb (*p* = 0.088) nor maternal Hb (*p* = 0.092) were significantly associated with birth weight. 

Finally, multivariate linear regression analysis with birth weight to placental weight ratio as the primary outcome is shown in Table 4. Only cord blood Hb (*p* = 0.001) and gestational age (*p* = 0.001) were predictors of placental efficiency, whereas maternal Hb levels were not (*p* = 0.631). For every unit (g/L) increase in cord blood Hb, the mean value of placental efficiency increased 0.015, while all other variables in the model were kept constant. The model explained 16% of the total variance in the model.

## 4. Discussion

Low maternal Hb levels are a frequent occurrence in pregnancy, in part due to blood volume expansion leading to hemodilution. Therefore, lower thresholds for anemia are used in pregnancy, corresponding to Hb levels of 100–110 g/L for mild, 70–99 g/L for moderate, and <70 g/L for severe anemia [25]. In the present study, more than 40% of women met the criterion for anemia—the majority with mild, and none with severe anemia. This finding is consistent with the notion that anemia is highly prevalent among pregnant women, even in industrialized countries [26]. The prevalence of anemia in pregnant women is due, in part, to the requirement for increased iron intake to sustain maternal blood volume expansion, as well as iron demands from the fetal-placental unit. Although information pertaining to dietary intake and, perhaps most importantly iron supplementation prior to and during pregnancy, was not collected herein, clinical studies using similar demographics (i.e., the Alberta Pregnancy Outcomes and Nutrition cohort) have reported low compliance with an intake of iron supplements (<30%) [27], which may contribute to the high prevalence of anemia. Notwithstanding, the rate of maternal anemia in the present study was higher than expected. One explanation may relate to the use of the HemoCue Hb201+ system for Hb assessments (see limitations section), which is routinely used at the Royal Alexandra Hospital as an option for immediate point of care measurement of Hb. Although the accuracy and precision of the Hemocue Hb201+ system has been validated, test results can differ depending on the method of blood collection. For example, Parker et al. showed that blood samples collected from a finger-prick using the HemoCue Hb201+ system may underestimate Hb levels (range (95% CI): −6, −1g/L) compared to venous samples assessed by automated hematology analyzers [28]. Nevertheless, all maternal samples in the present study were collected using the same method, enabling the relationships with placental weight and birth weights to be studied.

The primary objective was to assess whether maternal Hb levels or cord blood Hb levels more closely associate with placental weight. While low placental weight is associated with a risk of stillbirth (presumably reflecting inadequate placental surface area for nutrient and gas exchange), high placental weight is associated with neonatal morbidity and death, and this relationship remains even after controlling for birth weight [29]. Thus, placental weight is a powerful and independent determinant of pregnancy outcome. Maternal anemia has been shown to influence placental size at the time of delivery [3,4,10], although other studies have reported no such relationship [12,15,16,30]. In the present study, we found no correlation between maternal Hb status and placental weight. However, using cord blood Hb measurements as a surrogate for fetal Hb levels [22], we identified a robust inverse relationship with placental weights, even when adjusting for covariates. Likewise, lower cord blood Hb levels were associated with a higher placental to fetal weight ratio, which may be interpreted as an adaptation by the placenta to meet the demands of the fetus. The observation that birth weights were not correlated with cord blood Hb levels supports this hypothesis. Our findings suggest that measuring cord blood Hb levels, rather than maternal Hb levels, may provide important diagnostic information about in utero fetal adaptation to suboptimal placental function and neonatal health.

Due in part to the cross-sectional design of this study, it was not possible to elucidate whether cord blood Hb levels dictate placental size or vice versa. For example, in cases of reduced fetal Hb, placentas may be compensating for reduced oxygen-carrying capacity in fetal blood by growing larger, thereby providing a larger surface area for oxygen diffusion. In support of this possibility, oxygen is a potent regulator of placental growth and development [31,32], such that deficiencies in oxygen transport capacity may drive compensatory efforts by the placenta to optimize fetal growth. In animal models, the placenta responds to reduced oxygen availability and low maternal Hb by adapting its functional and structural phenotype, including increased vascularity, reduced diffusion barrier thickness, changes in zonal architecture and mitochondrial efficiency, and altered nutrient transport [33,34,35]. To better understand the effects of oxygen delivery on placental function, pregnancies at high altitudes are often studied as corollaries to low oxygen availability, without other confounding factors such as placental pathologies. In pregnant women residing at high altitudes, placental size and weights are generally larger in relation to fetal size, along with increased vascularity and decreased barrier thickness, all of which enhance the diffusion of more oxygen for fetal benefit [36,37]. It should be noted that high altitude causes increased maternal Hb production and oxygen-binding capacity [38], but the impact of high altitude on fetal Hb production and oxygen-binding has not been deduced.

An alternative explanation for the inverse relationship between cord blood Hb and placental size is that fetal Hb levels are dynamically regulated in proportion to placental size. Thus, a fetus can adapt to having a smaller placenta with reduced oxygen diffusion capacity by producing more Hb and in turn having relatively greater oxygen-carrying capacity. Additionally, elevated fetal Hb increases blood viscosity and slows placental hemodynamics (e.g., reduced umbilical vein flow), providing more time for oxygen diffusion in the placenta [39]. Placentas are adaptable organs, but only for transient periods during pregnancy. For example, in animals exposed to low oxygen atmospheres, the timing of hypoxia exposure is a critical determinant of placental adaptation. Generally, placentas of animals exposed to low oxygen in the first half of pregnancy adapt more effectively compared to animals exposed later in pregnancy because placental growth and vascularity are more dynamic in early pregnancy [40,41,42]. In contrast, new Hb can be produced over the course of several weeks to accommodate a new environment, such as humans newly adapting to high altitude conditions. Hb also has a long half-life (~120 days), so changes in Hb production can have a long-lasting impact on adaptation. Since the fetus depends on the placenta for its oxygen supply, fetal Hb may be produced in response to the oxygen diffusion capacity of the placenta to ensure optimal saturation in fetal blood.

In this study, we found that maternal Hb levels and cord blood Hb levels were correlated, albeit weakly. This positive correlation is consistent with some studies [19,20,21,22], but not others [23,24]. The relationship between maternal and cord blood Hb levels is complex, and may depend, at least in part, on certain intricacies of maternal-fetal iron transfer, as well as the etiology of anemia. In pregnancy, iron utilization by the fetus is prioritized over maternal demands [43], and therefore depletion of iron stores and anemia is likely to be more frequent and pronounced in the mother than the fetus (though it should be noted that our work in rats shows severe fetal anemia can occur with only mild maternal anemia [6,7]). Moreover, a third of all cases of anemia are not due to iron deficiency [44] but are attributed to complications such as maternal alloimmunization, infection/inflammation, and other nutritional deficiencies (folate, vitamin B12); these cases may not necessarily involve a concomitant reduction of maternal and fetal Hb. Irrespective of the underlying cause, the weak correlation confirms that maternal Hb status is not a reliable means of identifying fetal/neonatal anemia.

Several studies have investigated the relationship between maternal anemia and placental weight (see Introduction), but the relationship between cord blood Hb status and placental weight has not been investigated. The present study enabled us to study these relationships in a relatively healthy population where confounders (e.g., infection, malnutrition) of Hb status are likely minimized. Moreover, given that the majority of women recruited for this study exhibited either normal Hb levels or only mild anemia, the results described herein are likely applicable to a large proportion of pregnant women.

There are several limitations in this study that warrant discussion. First, placental function cannot be accurately modeled by measuring weight alone. Other factors, such as placental vascularization, barrier thickness, volume, and hemodynamics are important considerations. Notwithstanding, placental weight provides useful information, and due to its ease of collection, this study represents an important first step in elucidating placental adaptation to anemia. The ratio of birth weight to placental weight was also analyzed herein, because studies have used this relationship as a proxy for placental efficiency and investigated its relationship with the risk of chronic disease in the offspring (reviewed in ref. [45]). However, the appropriateness of this ratio as a measure of placental adaptation has been questioned, particularly in clinical studies [46,47]. For this reason, care was taken not to over-interpret these data, and further studies investigating the relationship between maternal and cord blood Hb levels with metrics of placental function are needed.

Second, this study was limited to maternal and cord blood Hb determination in singletons delivered by cesarean section. By confining the analysis to elective cesarean sections at term, we avoided potential confounding variables such as labored delivery and complications associated with prematurity. However, women who undergo an elective cesarean section, on average, are older than the general population of delivering women [48,49], and since elective cesarean sections are seldom performed to deliver preterm infants, future studies should be conducted using a more inclusive cohort of pregnant women to determine the applicability of our findings to a larger patient demographic. The issue with preterm births is particularly important because although cord blood Hb was correlated with placental weight at term, whether this trend holds true earlier in pregnancy (e.g., during the second trimester) is not presently clear. Placentation is inherently more adaptable early in pregnancy when placental growth and vascularization are robust. Thus, low Hb levels early in pregnancy may promote early adaptive and beneficial changes, whereas the placenta may not be able to adapt as effectively to altered Hb levels during later stages of pregnancy. As such, the timing of altered Hb levels and the resulting consequences on placentation should be explored in more detail. Third, placental weights presented herein represent untrimmed weights, which may explain the higher average values than those reported in the literature. Although the inclusion of umbilical cord and membranes contribute to an increased weight, Leary et al. showed that trimmed and untrimmed placental weight were highly correlated [50], suggesting that the relative weight of membranes trimmed is generally proportional to placental weight. Finally, some important information (e.g., parity) was not recorded, which could have profound effects on placental size [51].

## 5. Conclusions

The results presented herein suggest that cord blood, which is routinely collected at birth, could be analyzed to identify anemia in the neonate and provide insight into placental adaptations during pregnancy. This information, particularly cord blood Hb levels, could inform intervention strategies to mitigate the deleterious outcomes associated with iron deficiency and anemia in newborns and infants. Further studies are required to establish mechanisms through which fetal Hb and placental weight synchronize to ensure optimal oxygen delivery to the fetus.

## Figures and Tables

**Table 1:** Study population characteristics (*n* = 182).

Variable	Mean (Range)	SD
Maternal age (y)	32.5 (20.0–45.5)	5.1
Maternal height at delivery (cm)	163 (118–185)	8
Maternal Hb (g/L)	111.5 (75.0–146.0)	14.0
ASA (n, %)		
Level 1	84 (46%)	
Level 2	90 (49%)	
Level 3		8 (4%)
Cord blood Hb (g/L)	142.3 (100.0–191.0)	15.9
Birth weight (g)	3458 (2400–5080)	505
Gestational age (wk)	38.6 (35.0–41.0)	1.0
Placental weight (g)	772.3 (395.0–1500.0)	188.4
Infant sex (n, %)		
Male	85 (46%)	
Female	94 (54%)	
APGAR Score		
1 min	9.0 (4.0–10.0)	1.1
5 min	9.0 (5.0–10.0)	0.5

ASA, American Society of Anesthesiologists physical status classification; Hb, hemoglobin; SD, standard deviation.

**Table 2:** Multiple linear regression analysis of placental weight (*r*^2^ = 0.38).

Variable	*β*	SE	95% CI	*p*
Birth weight	0.224	0.025	0.173, 0.275	<0.001
Cord blood Hb	−2.444	0.741	−3.908, −0.982	0.001
Gestational age	−39.745	13.869	−67.121, −12.370	0.005
ASA	37.876	20.490	−2.568, 78.321	0.066
APGAR (5 min)	36.726	21.994	−6.686, 80.139	0.097
Maternal age	−2.330	2.275	−6.822, 2.161	0.307
Maternal Hb	0.660	0.831	−0.980, 2.300	0.428
Sex of newborn	−0.948	22.966	−46.279, 44.383	0.967
Maternal height	−0.018	1.480	−2.939, 2.903	0.990

ASA, American Society of Anesthesiologists physical status classification; β, regression coefficient; CI, confidence interval; Hb, hemoglobin; SE, standard error.

**Table 3:** Multiple linear regression analysis of birth weight (*r*^2^ = 0.51).

Variable	*β*	SE	95% CI	*p*
Gestational age	217.108	29.886	158.116, 276.100	<0.001
Placental weight	1.168	0.163	0.845, 1.490	<0.001
Maternal height	6.155	3.520	−0.794, 13.104	0.082
Cord blood Hb	3.152	1.840	−0.480, 6.784	0.088
Maternal Hb	−3.353	1.978	−7.260, 0.552	0.092
Sex of newborn	−59.904	55.366	−169.193, 49.385	0.281
Maternal age	4.844	5.461	−5.935, 15.624	0.376
APGAR (5 min)	−43.964	53.043	−148.668, 60.740	0.408
ASA	1.493	52.427	−101.994, 104.980	0.977

ASA, American Society of Anesthesiologists physical status classification; β, regression coefficient; CI, confidence interval; Hb, hemoglobin; SE, standard error.

**Table 4:** Multiple linear regression analysis of birth weight to placental weight ratio (*r*^2^ = 0.16).

Variable	*β*	SE	95% CI	*p*
Cord blood Hb	0.015	0.004	0.007, 0.024	0.001
Gestational age	0.247	0.074	0.101, 0.393	0.001
APGAR (5 min)	−0.168	0.131	−0.426, 0.091	0.091
Maternal age	0.017	0.014	−0.010, 0.044	0.207
ASA	−0.139	0.130	−0.395, 0.117	0.286
Maternal Hb	−0.002	0.005	−0.012, 0.007	0.631
Sex of newborn	0.023	0.137	−0.248, 0.295	0.864
Maternal height	0.001	0.009	−0.016, 0.018	0.885

ASA, American Society of Anesthesiologists physical status classification; β, regression coefficient; CI, confidence interval; Hb, hemoglobin; SE, standard error.

## Data Availability

The datasets used and/or analyzed during the current study are available from the corresponding author on reasonable request.
